# [Retracted] Anti-tumor peptide AP25 decreases cyclin D1 expression and inhibits MGC-803 proliferation via phospho­extracellular signal-regulated kinase-, Src-, c-Jun N-terminal kinase- and phosphoinositide 3­-kinase-associated pathways 

**DOI:** 10.3892/mmr.2026.13950

**Published:** 2026-06-29

**Authors:** Jialiang Hu, Tao Cheng, Lijun Zhang, Beicheng Sun, Lei Deng, Hanmei Xu

Mol Med Rep 12: 4396–4402, 2015; DOI: 10.3892/mmr.2015.3912

Following the publication of the above paper, it was drawn to the Editor's attention by a concerned reader that the following anomalies/potential areas of concern appeared to be associated with the western blot data featured in Figs. 2, 4 and 6 (including the description of additional features that came to light based upon an independent analysis of the data in this paper that was undertaken by the Editorial Office):

i) There were overlapping data bands comparing the cyclin D1 and β-actin bands in Fig. 2C and D with those in Fig. 4B, where the figure legends indicated that the experimental conditions for these figures were different; and ii) in Fig. 6A, two pairings of cyclin D1 blots appeared to be duplicated/doubled up (giving rise to four sets of blots), and moreover, the associated β-actin blots also appeared to exhibit different overlaps of individual bands/pairs of bands.

Given the identification of so many instances of data duplication comparing both within and between the abovementioned figures in this paper, the Editor has decided that this paper should be retracted from the Journal on account of a lack of confidence in the data. The authors were asked for an explanation to account for these concerns, but the Editorial Office did not receive a reply. The Editor apologizes to the readership for any inconvenience caused.

